# MicroRNA Expression in Alzheimer Blood Mononuclear Cells

**DOI:** 10.4137/grsb.s361

**Published:** 2007-12-20

**Authors:** Hyman M. Schipper, Olivier C. Maes, Howard M. Chertkow, Eugenia Wang

**Affiliations:** 1 Department of Neurology and Neurosurgery, McGill University, Montreal, Canada; 2 Department of Medicine (Geriatrics), McGill University, Montreal, Canada; 3 Centre for Neurotranslational Research, Lady Davis Institute for Medical Research, Sir Mortimer B. Davis Jewish General Hospital, Montreal, Canada, H3T1E2; 4 Bloomfield Centre for Research in Aging, Lady Davis Institute for Medical Research, Sir Mortimer B. Davis Jewish General Hospital, Montreal, Canada, H3T1E2; 5 Gheens Center on Aging and Department of Biochemistry and Molecular Biology, School of Medicine, University of Louisville, Louisville, Kentucky, U.S.A., 40202

**Keywords:** Alzheimer disease, gene expression, microarray, neurodegeneration, noncoding small RNA

## Abstract

Various coding genes representing multiple functional categories are downregulated in blood mononuclear cells (BMC) of patients with sporadic Alzheimer disease (AD). Noncoding microRNAs (miRNA) regulate gene expression by degrading messages or inhibiting translation. Using BMC as a paradigm for the study of systemic alterations in AD, we investigated whether peripheral miRNA expression is altered in this condition. MicroRNA levels were assessed using the microRNA microarray (MMChip) containing 462 human miRNA, and the results validated by real time PCR. Sixteen AD patients and sixteen normal elderly controls (NEC) were matched for ethnicity, age, gender and education. The expression of several BMC miRNAs was found to increase in AD relative to NEC levels, and may differ between AD subjects bearing one or two APOE4 alleles. As compared to NEC, miRNAs significantly upregulated in AD subjects and confirmed by qPCR were miR-34a and 181b. Predicted target genes downregulated in Alzheimer BMC that correlated with the upregulated miRNAs were largely represented in the functional categories of Transcription/Translation and Synaptic Activity. Several miRNAs targeting the same genes were within the functional category of Injury response/Redox homeostasis. Taken together, induction of microRNA expression in BMC may contribute to the aberrant systemic decline in mRNA levels in sporadic AD.

## Introduction

MicroRNA are noncoding small RNA that bind target sites either in the 3′ untranslated region (UTR) of mRNA to inhibit translation or at the coding regions to degrade the messages (Lee et al. 1993; Ruvkun et al. 2004; Ambros, 2004; Lim et al. 2005). Mature microRNAs (miRNA) are approximately 22 nucleotides in length and are expressed under the control of an RNA polymerase II promoter. The catalogue of human miRNA has expanded substantially in the last few years and the number of miRNA species is predicted to be close to one thousand (Hammond, 2006). Loss-of-function mutations have greatly facilitated elucidation of the biological functions of miRNA (Ambros, 2004) including their roles in development, cell differentiation (Ambros, 2004; Ouellet et al. 2006), lifespan (Boehm and Slack, 2005) and in diseases such as cancer (Hammond, 2006) and neurodegeneration (Bilen et al. 2006a). In mammals, neural tissues are believed to manifest the most complex and specific miRNA expression patterns relative to other organs (Babak et al. 2004; Strauss et al. 2006).

Alzheimer disease (AD) is a dementing illness characterized by progressive neuronal degeneration, gliosis, and the accumulation of intracellular inclusions (neurofibrillary tangles) and extracellular deposits of amyloid (senile plaques) in discrete regions of the basal forebrain, hippocampus, and association cortices (Chertkow et al. 2001; Selkoe, 1991). The etiology of sporadic AD is likely multifactorial, with carriage of the apolipoprotein E ɛ4 (APOE4) allele constituting a strong risk factor for the development of this condition (Kamboh, 2004).

Gene expression studies in AD have shown substantial downregulation of various mRNA species in brain (Pasinetti, 2001), peripheral blood mononuclear cells (BMC) (Maes et al. 2006) and lymphocytes (Scherzer et al. 2004) relative to non-demented control values. Furthermore, numerous investigations have implicated impairment of protein synthesis in AD tissues (Keller, 2006) and diminished concentrations of specific proteins in the cerebrospinal fluid (CSF) of these patients (Puchades et al. 2003). In a small study of 13 miRNAs, levels of miR-9, miR-125b, and miR-128 were found to be increased in human AD hippocampus relative to control values (Lukiw, 2007). In light of the above, we hypothesized that augmented miRNA expression may be responsible for the suppression of specific mRNA species both in AD brain and peripheral tissues. To test this hypothesis in the latter, we screened 462 human miRNA (from let-7 family to miR-663) in BMC derived from well-characterized cases of mild sporadic AD and age-matched normal elderly control subjects and, based on predicted miRNA targets, ascertained whether the accruing data may account for the patterns of mRNA downregulation in Alzheimer BMC previously reported by our laboratories (Maes et al. 2006).

## Materials and Methods

### Subjects

This study was approved by the Research Ethics Committee of the Sir Mortimer B. Davis Jewish General Hospital (JGH). Written informed consent was obtained from all patients or their primary caregivers. Recruited patients with sporadic AD were assessed by a neurologist or geriatrician at the JGH-McGill University Memory Clinic, a tertiary care facility for the evaluation of memory loss in Montreal. All AD subjects underwent formal neuropsychological testing as previously described (Chertkow et al. 2001; McKhann et al. 1984). Normal elderly controls (NEC) were recruited from Family Practice Clinics at the JGH. The latter scored within one SD of age- and education-standardized normal values on a series of memory and attention tests. The Mini-Mental State Examination (MMSE) (Folstein et al. 1975) was administered to all subjects. Subjects with chronic metabolic and inflammatory conditions or acute illness were excluded from the study. Student’s unpaired t-test was performed to assess statistical differences in age, years of formal education and MMSE scores between groups. Apolipoprotein E genotyping was performed as previously described (Maes et al. 2006). APOE4-positive persons were defined as individuals bearing one or two APOE4 alleles.

### Blood samples and extraction

Whole blood was collected and BMC were isolated as previously described (Maes et al. 2006). The cell pellet was lysed in Trizol (Invitrogen, Canada) and immediately stored at −80 °C until further processing. Extraction of RNA was performed as described by Lacelle et al. 2002 (Lacelle et al. 2002). Briefly, for 1 mL of Trizol, 0.2 mL of chloroform was added and mixed for 15 seconds. After 3 minutes incubation at room temperature (RT), the samples were centrifuged at 12,000 g (4 °C) for 15 min. The upper aqueous layer containing RNA was transferred to another microcentrifuge tube for RNA extraction. Total RNA was solubilized in DEPC-treated water and purified using RNeasy Mini columns (Qiagen, Canada) according to manufacturer instructions.

Small RNA enrichment was performed according to Park et al. 2002 (Park et al. 2002). Total RNA samples were adjusted to 400 μL with RNase-free water and then 50 uL of NaCl (5M) and 50 uL PEG 8000 (v/v 50%) were added. The sample was incubated on ice for 2 hours, and centrifuged for 10 minutes at 13 000 rpm (4 °C). Supernatant containing small RNA was transferred to a microcentrifuge tube and 50 uL of sodium acetate (3M, pH 4.6) and 1 mL of 100% ethanol were added. The samples were mixed and incubated at −20 °C for 2 hours and centrifuged for 10 minutes at 12 000 g (4 °C). The supernatant was discarded, and the pellet was washed with 1 mL of cold 75% ethanol and, centrifuged for 10 minutes at 12 000 g (4 °C). The pellet was dried and dissolved in 12 μL of RNase-free water at 60 °C for 10 minutes. Nucleic acid concentrations were determined at 260 nm by spectrophotometry and samples were stored at −80 °C.

### MicroRNA profiling

We applied rigorous selection criteria on samples to control for ethnicity, health status, age and education. Only samples with good yields in total RNA and small RNA enrichment were used (Supplementary Table 1). Small RNA samples were labeled with digoxigenin (DIG) at the 3′ end using the DIG Oligonucleotide Tailing Kit, 2nd Generation (Roche Diagnostics, U.S.A.). One μg of small RNA was labeled in a total volume of 20 μL as described by Wang et al. (Wang et al. 2002). The human microRNA microarray (MMChip) consisted of 462 human anti-sense DNA sequences of microRNAs obtained from miRBase (http://microrna.sanger.ac.uk/) and spotted on nitrocellulose membrane as described (Wang et al. 2002). Microarray chips were pre-hybridized in 1 mL of DIG Easy Hyb solution (Roche Diagnostics, U.S.A.) at 42 °C for 1 hr. DIG-labeled small RNAs from one human subject were added to the membrane and hybridization was performed at 42 °C for 16 hrs. The membrane was washed twice (5 min) in solution 1 (2 × SSC, 0.1% SDS) at RT, incubated for 20 minutes at 37 °C in solution 2 (0.5 × SSC, 0.1% SDS), and 5 minutes in 1× maleic acid solution (100 mM maleic acid, 150 mM NaCl, pH 7.5) at RT. The membrane was blocked in 1.5% Blocking reagent (w/v; Roche Diagnostics, USA). The membrane was incubated for 30 minutes with 1:1500 Anti-Digoxigenin-AP Fab fragments (Roche Diagnostics, U.S.A.). The miRNAs hybridized on the MMchip were revealed by the alkaline phosphatase chromogenic reaction of NBT/BCIP dye as per manufacturer’s instructions (Roche Diagnostics, U.S.A.).

Only MMchips with excellent hybridization intensities were analyzed. Hybridization intensities were measured using an Expression 1680 scanner (Epson, U.S.A.) and data acquired using Array-Pro Analyzer 4.5 software (Media Cybernetics, MD, USA). Net intensity was derived from whole cell area measurement and corrected using mean intensity of ring background of surrounding spots. Microarray data analyses were performed with SAM software, version 3.02 (Significance Analysis of Microarrays, Stanford University, CA, U.S.A.). The variable “Block” was used to account for the experimental batches (block effect). Kolmogorov—Smirnov statistics were generated by Gene Set Enrichment Analysis (GSEA) software (Subramanian et al. 2005).

Hierarchical clustering analysis was performed using GenePattern software (www.broad.mit.edu/cancer/software/genepattern/; Broad Institute, MA, U.S.A.) Functional attribution of correlated gene targets was made according to the SOURCE database (http://source.stanford.edu) and Gene Ontology Tree Machine (http://bioinfo.vanderbilt.edu/gotm).

### QRT-PCR validation

For real time PCR validation, 0.1 μg of small RNA were quantified using the NCode qRT-PCR kit (Invitrogen, U.S.A.). Mature DNA sense sequences of tested miRNAs were used as forward PCR primers. 5S rRNA served as reference gene, and was probed using an internal forward primer (CAGGGTCGGGCCTGGTTAGTACTTG).

For the miRNA let-7f, the qRT-PCR was performed using the TaqMan MicroRNA Reverse Transcription kit, TaqMan MicroArray assay (hsa-let-7f, RT 382) and TaqMan Fast Universal PCR (NoAmpErase UNG;Applied Biosystems, U.S.A.) with 1 ng of small RNA. The TaqMan specific primer U24 small nucleolar RNA (RNU24, RT 1001) was used as reference gene.

All real time PCR reactions were performed on a 7500 Fast System Real Time PCR cycler (Applied Biosystems, U.S.A.) according to manufacturer’s instructions. MicroRNA fold changes between diagnostic groups or genders were calculated by the delta Ct method.

## Results

### MicroRNA expression in Alzheimer BMC

Mean ages between the NEC (76 ± 6 years) and AD (78 ± 5 years) groups were not significantly different (p = 0.26; Supplemental Table 1). Subjects in the AD group had fewer years of formal education (12.6 years as compared to 15 years for NEC, p = 0.05) and scored significantly lower on the MMSE (23/30 as compared to 29/30 for NEC, p < 0.0001). Yields in small RNA enrichment did not significantly differ between NEC and AD samples (p = 0.95).

**Figure 1:**
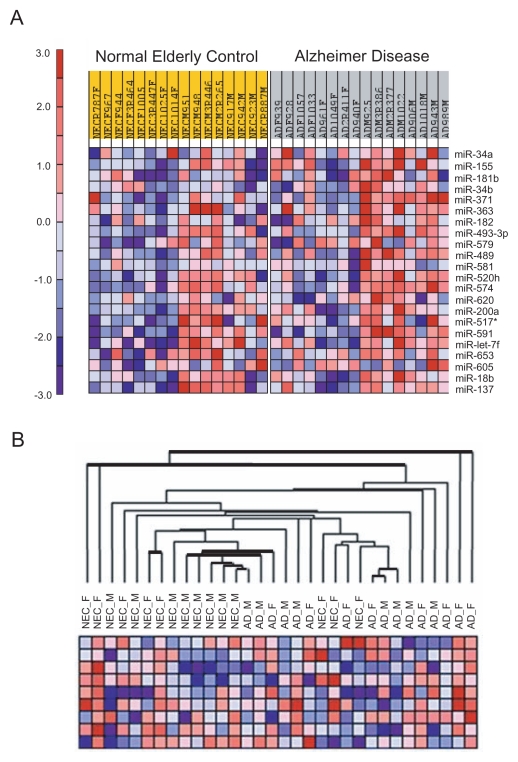
Gene set enrichment analysis and hierarchical clustering of microRNA expression in Alzheimer BMC. (**A**) The significantly upregulated miRNA in AD are ranked in order of significance from top to bottom. (**B**) The hierarchical clustering of subjects was determined using Pearson correlation.

The study consisted of 16 NEC and 16 AD miRNA expression profiles, analyzed in four independent experimental blocks with equal numbers of women and men. Only upregulated miRNA expression was found to be significantly altered in Alzheimer BMC by T-statistics (Table 1). The relative increases in miRNA in Alzheimer BMC were modest, in the range of 1.1 to 1.4-fold. Significant microarray data analyzed by SAM (Table 1) was subsequently ranked by Kolmogorov—Smirnov statistics using the Gene Set Enrichment Analysis (GSEA) software (Subramanian et al. 2005). The enrichments of the significant upregulated miRNA were confirmed for miR-34a and 181b. (Fig. 1A). Although miR-155 was ranked in second position, the fold increase in AD was not validated by quantitative PCR (see below).

We observed slightly different levels of miRNA expression between male and female BMC as illustrated by the heat map generated by GSEA (Fig. 1A), but the differences analyzed by SAM were not significant (data not shown). Next, we stratified NEC and AD miRNA signatures according to APOE4 status (Table 2). Similarly to the entire cohort, we found common miRNA upregulated in AD, with tendencies for over-representation of certain miRNA in the APOE4-negative (i.e. miR-34a, 517*, let-7f, 200a), and possibly the APOE4-positive (i.e. miR-371; miR-181b) strata.

We next performed a higher order analysis of the cohort’s miRNA expression signatures by hierarchical clustering using Pearson correlations (Fig. 1B). An apparent classification of NEC and AD was obtained, although three female NEC signatures subgrouped with AD miRNA profiles. The complexity of the different levels of clusters may be related to the inherent experiment block effect existing between the MMchips. Sub-grouping for gender or using the significantly upregulated miRNAs of Table 1 did not improve the classifications (data not shown).

### Real time PCR validation

**Figure 2:**
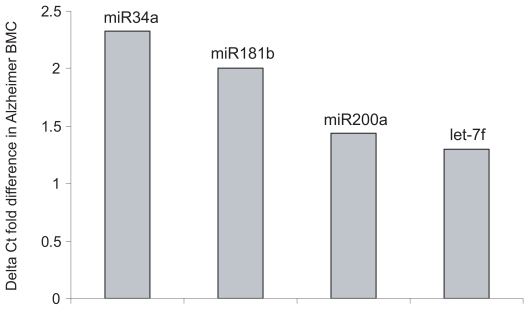
Quantitative real time PCR of miRNA levels in AD. Fold differences in miRNA levels between NEC and AD were estimated by the delta Ct method.

The above comparison for the whole cohort was validated by real time PCR. Levels of selected miRNAs in 10 samples (NEC and AD) were determined by qRT-PCR, and the average Ct values were used to estimate the difference in expression levels between diagnostic groups (Fig. 2).

Of the five selected miRNAs tested by qPCR, only one miRNA (miR-155) was not validated. Importantly, the delta Ct fold differences estimated in AD for miR-34a, 181b, 200a and let-7f corresponded to their order of significance reported in Table 1. The qPCR data suggest that the increase in miRNA levels in AD may be underestimated in the microarray platform (Table 1).

### Target predictions

Target predictions for the significantly upregulated miRNA in Alzheimer BMC ascertained from the miRBase (Sanger Institute, http://microrna.sanger.ac.uk/targets/v4/) were compared with the down-regulated mRNAs previously reported in Alzheimer BMC (Maes et al. 2006). Of the predicted hundreds of targets for any specific miRNA, a range of 10 to 30 downregulated transcripts in Alzheimer BMC were correlated, however only transcripts with significantly lower levels in AD are reported in Table 3. The percentage of targets per miRNA is reported in Table 1. Interestingly, miR-181b, which exhibited the greatest fold-increase expression in Alzheimer BMC, targeted the highest proportion of downregulated genes in AD BMC (31.1%), followed by miR-34a (21.7%). In contrast, targets of miR-200a correlated least with the genomic data (7.5%). Target genes with significantly downregulated transcript levels in AD frequently correlated with miR-181b (NDUFS3, HSF2), let-7f (HERPUD1, TBPL1) and miR-34a (HNRPR, BTF3).

**Figure 3:**
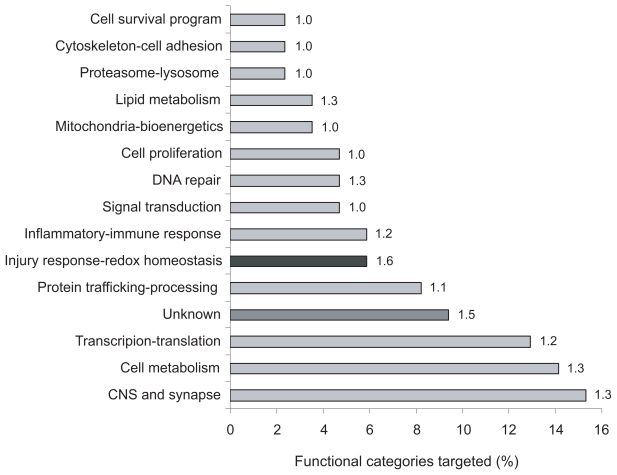
Functional categories of predicted microRNA targets downregulated in Alzheimer BMC. Targets of upregulated BMC miRNA were compared to previous downregulated mRNA data in Alzheimer BMC (Maes et al. 2006), and summarized according to percentage of targets per functional category. The average number of miRNAs targeting the same gene within each category is reported.

The functional categories for these putative targets are summarized in Figure 3. Interestingly, genes with multifunctional roles in Synapse Activity represented the most extensively targeted category. The enriched GO categories specified by GOTREE for the targeted downregulated genes were: Transcription (P = 0.001), Protein Transport (P = 0.006), and the Peroxisome (P = 0.005). The highest number of individual miRNAs targeting a common gene within the same functional category corresponded to Injury Response/Redox Homeostasis. MicroRNAs let-7f, 34a 181b and 200a shared common targets in several functional categories (Table 3).

The enriched GO categories for the downregulated targets identified for some of the individual upregulated miRNAs were as follows: Cell Homeostasis and Peroxisome for miR-34a; Cell Cycle and DNA Damage for miR-181b; and Vesicle Processes for miR-517*. Represented GO categories were also associated with miRNAs, such as: Cell Cycle with miR-200a and DNA Damage with miR-517*.

## Discussion

Our study was based on the same population described in a previous transcriptome analysis (Maes et al. 2006) in order to compare microRNA action and their target genes at the message level. We made a stringent sample selection based on: (1) well-ascertained cases and controls matched for ethnicity, gender and age and (2) excellent total RNA quality and yield of purified microRNA for the screening of 462 currently-known human microRNA species. We found that human BMC expressed a broad range of miRNAs, representing 20% of the 462 miRNA spotted on nitrocellulose membranes in the array format (Supplemental Table 2).

MicroRNA expression profiles have shown greater accuracy in classifying cancers than have gene expression profiles (Lu et al. 2005). Although our classification of miRNA expression signatures may have been affected by experimental variations, we obtained relatively good discrimination of NEC vs. AD cases. However, the complexity and variability of miRNA expression observed between subjects could have also arisen from the diversity of cell types comprising the BMC fraction (Jison et al. 2004). It still remains to be determined whether the miRNA signature of a specific blood mononuclear cell type would be more effective in the classification of neurodegenerative diseases.

Despite the heterogeneity of BMC, we observed a significant increase in the expression of miR-34a and 181b in mild sporadic AD as compared to age-matched normal controls. A higher upregulation of miR-181b in Alzheimer BMC may also occur in APOE4-positive AD subjects. However, the higher FDR of 22% and low number of APOE4-positive chips in the NEC group does not allow us to determine conclusively the impact of APOE4 status on miRNA expression. Nevertheless, the APOE4 allele is associated with an earlier onset of the disease (Hsiung et al. 2004), and higher expression of miRNAs could occur in these subjects as suggested by our results. If validated, induction of miRNA expression may provide a surrogate marker of AD progression.

In this regard, it is interesting to note that a reduction in dicer activity promotes tau toxicity in Drosophila (Bilen et al. 2006b). Thus, certain hallmark neuropathological features of AD may represent events of dysregulated miRNA processing. A reduction of miRNA processivity should instead lead to a decrease in miRNA levels. In fact, increases in miRNAs levels were reported in human AD hippocampus (Lukiw, 2007), and this possible paradox in humans should be addressed in future investigations. However, the miRNAs observed in Alzheimer BMC differed from the few miRNAs screened in the latter study. In both studies, induction of specific miRNAs may contribute to the suppression of multiple mRNA species and thereby impact a host of cellular mechanisms (Lukiw, 2007). Moreover, concomitant induction of several miRNA species may act in an additive or synergistic manner to inhibit gene expression (Krek et al. 2005). In fact, studies have determined that individual microRNAs can downregulate several mRNA species (Lim et al. 2005) and induce mRNA instability (Sood et al. 2006).

The 3′ UTR of target genes and the cellular context strongly influence the action of miRNA (Didiano and Hobert, 2006), and our study provides insights leading to the identification of probable miRNA targets in human BMC. We discerned a prevalence of putative downregulated genes in the functional categories of Synapse Activity, Transcription, and Injury/Redox Homeostasis. Importantly, Cell Cycle and DNA Damage related GO categories are targeted by miR-181b, 200a, and 517*.

Taken together, these observations further support our model linking the development of AD pathology to systemic dysfunction in the cellular stress/antioxidant response and genomic maintenance (Maes et al. 2006). These data are commensurate with reports of augmented oxidative DNA and RNA damage and deficient transcription and translation in AD brain and peripheral tissues (Markesbery and Lovell, 2006; Shan and Lin, 2006). The latter impairments may, in turn, contribute to the cytoskeletal abnormalities and neurofibrillary degeneration characteristic of AD-affected neural tissues (Maes et al. 2006).

Caution must be exercised in extrapolating from BMC miRNA data sets to gene expression profiles in AD brain given the different 3′ UTR regions inherent to neuronal mRNA relative to peripheral tissues (Sood et al. 2006). A wide array of miRNA species should be surveyed in AD-affected brain tissue to determine whether miRNA dysregulation exists therein comparable to that observed in the current Alzheimer BMC study.

The current study provides, to our knowledge, first evidence of augmented microRNA expression in Alzheimer BMC, as well as the framework for future miRNA-target experiments on altered cellular functions related to this disease. For example, miRNAs may account for the suppression of mRNA species implicated in the cellular stress response and DNA repair previously reported in Alzheimer BMC (Maes et al. 2006). Dysregulation of BMC miRNA in sporadic AD may shed new light on the pathogenesis of AD and possibly provide useful diagnostic/prognostic biomarkers of this common affliction.

## Supplemental Materials

Supplemental Table 1 Demographics of cohort and small RNA yields.

**Table:** 

Diagnosis	Subject ID	Gender	Age	Education	MMSE	APOE	Total RNA (μg)	Small RNA (μg)	Ratio ^a^ (%)
NEC	967	F	80	11	29	2,3	41.7	1.2	2.9
NEC	944	F	68	18	30	3,3	80.4	1.7	2.1
NEC	1014	F	83	21	29	3,3	73.6	2.1	2.8
NEC	3R447	F	78	17	29	3,3	23.5	1.1	4.5
NEC	3R464	F	78	10	28	3,3	115.8	1.2	1.0
NEC	R787	F	87	15	30	3,3	26.8	1.7	6.5
NEC	1005	F	68	15	30	3,4	97.0	1.2	1.3
NEC	1025	F	71	15	28	3,4	51.1	3.4	6.6
NEC	917	M	72	15	29	3,3	23.5	1.8	7.7
NEC	923	M	84	12	27	3,3	43.2	1.0	2.2
NEC	942	M	72	11	29	3,3	57.8	1.9	3.3
NEC	948	M	80	16	30	3,3	34.9	1.4	4.1
NEC	R887	M	74	20	30	3,3	56.4	3.1	5.5
NEC	951	M	80	16	30	3,4	15.1	1.1	7.0
NEC	R265	M	73	22	30	3,4	44.3	1.0	2.3
NEC	3R446	M	71	9	29	4,4	62.9	1.7	2.6
AD	940	F	75	13	21	3,4	52.9	1.1	2.1
AD	961	F	79	10	23	3,4	100.1	1.8	1.8
AD	939	F	78	6	27	3,3	76.9	1.1	1.4
AD	2R411	F	80	14	24	3,3	38.4	2.6	6.8
AD	1049	F	84	16	24	2,4	18.9	2.6	13.9
AD	1057	F	72	11	27	3,4	37.6	1.1	2.8
AD	928	F	81	13	13	2,2	39.4	1.1	2.9
AD	1033	F	88	12	nd	3,3	48.0	0.9	1.9
AD	906	M	73	14	25	3,3	28.1	1.3	4.7
AD	925	M	80	15	28	3,3	52.1	1.4	2.6
AD	943	M	75	20	17	3,3	77.8	1.6	2.0
AD	989	M	69	11	21	4,4	36.7	0.9	2.6
AD	1018	M	76	12	23	4,4	34.3	2.8	8.1
AD	1022	M	84	12	22	3,4	61.0	2.5	4.1
AD	2R377	M	81	15	26	3,4	117.0	1.3	1.1
AD	3R386	M	79	7	25	3,3	73.8	1.8	2.5

a Percentage of small RNA over total RNA.

Supplemental Table 2 miRNA expression in elderly human blood mononuclear cells.

**Table:** 

High (intensity 20–8) ^a^.	Medium (intensity <7)	Low (intensity <3)
hsa-let-7c	hsa-let-7g	hsa-let-7i
hsa-let-7f	hsa-miR-34a	hsa-miR-10a
hsa-miR-10b	hsa-miR-34b	hsa-miR-18b
hsa-miR-18a	hsa-miR-92b	hsa-miR-23b
hsa-miR-27b	hsa-miR-125b	hsa-miR-26a
hsa-miR-95	hsa-miR-136	hsa-miR-93
hsa-miR-137	hsa-miR-181b	hsa-miR-107
hsa-miR-188	hsa-miR-182	hsa-miR-146a
hsa-miR-200b	hsa-miR-195	hsa-miR-148a
hsa-miR-373*	hsa-miR-200a	hsa-miR-152
hsa-miR-376a*	hsa-miR-219	hsa-miR-155
hsa-miR-377	hsa-miR-373	hsa-miR-192
hsa-miR-380-5p	hsa-miR-489	hsa-miR-363
hsa-miR-509	hsa-miR-515-5p	hsa-miR-371
hsa-miR-510	hsa-miR-518a	hsa-miR-424
hsa-miR-517*	hsa-miR-520b	hsa-miR-431
hsa-miR-520h	hsa-miR-539	hsa-miR-449b
hsa-miR-523	hsa-miR-548b	hsa-miR-493-3p
hsa-miR-551a	hsa-miR-562	hsa-miR-513
hsa-miR-561	hsa-miR-577	hsa-miR-569
hsa-miR-574	hsa-miR-579	hsa-miR-575
hsa-miR-582	hsa-miR-600	hsa-miR-581
hsa-miR-585	hsa-miR-607	hsa-miR-587
hsa-miR-591	hsa-miR-620	hsa-miR-605
hsa-miR-598	hsa-miR-623	hsa-miR-608
hsa-miR-603	hsa-miR-624	hsa-miR-638
hsa-miR-609	hsa-miR-627	hsa-miR-652
hsa-miR-612	hsa-miR-646	
hsa-miR-621	hsa-miR-647	
hsa-miR-633	hsa-miR-653	
hsa-miR-641	hsa-miR-661	
hsa-miR-649		
hsa-miR-659		
hsa-miR-660		

a Hybridization intensity after DIG immunodetection (see Methods section).

## Figures and Tables

**Table 1:** Upregulated microRNA in Alzheimer BMC.

MicroRNA	Fold change	Score (d)	q-value (%)	BMC targets (%)^a^
hsa-miR-34a	1.2	1.3	0.0	21.7
hsa-miR-579	1.2	1.2	0.0	12.3
hsa-miR-181b	1.4	1.1	0.0	31.3
hsa-miR-520h	1.1	1.0	0.0	-
hsa-miR-155	1.2	0.9	5.1	-
hsa-miR-517*	1.1	0.9	5.1	11.3
hsa-let-7f	1.1	0.8	5.1	16.0
hsa-miR-200a	1.1	0.7	5.1	7.5
hsa-miR-371	1.1	0.7	5.1	-

False discovery rate (q-values) was 5.1% of 16 significant miRNAs.

a Predicted targets were determined for miRNAs having scores (d) higher than 1.0 or 0% q-values in both Tables 1 and 2.

**Table 2:** Altered miRNA expression in APOE4-negative and -positive strata

APOE4 status	MicroRNA	Fold change	Score (d)	q-value (%)
negative	hsa-miR-34a	1.4	1.8	0.0
negative	hsa-miR-517*	1.2	1.7	0.0
negative	hsa-let-7f	1.2	1.5	0.0
negative	hsa-miR-200a	1.2	1.4	0.0
positive	hsa-miR-371	1.4	1.1	22.2
positive	hsa-miR-181b	1.7	1.0	22.2

False discovery rates (q-values) were 0% in the negative stratum and 22.2% in the positive stratum of 4 and 4 significant miRNAs respectively.

**Table 3:** Predicted miRNA targets downregulated in Alzheimer BMC.

Upregulated microRNA	Target^a^	Target Gene Name	Fold change	Score (d)	Function
Cell development and metabolism					
181b	AHCY	S-adenosylhomocysteine hydrolase	0.46	−1.14	methylation/homocysteine
34a	BCKDK	branched chain alpha- ketoacid dehydrogenase kinase	0.56	−1.91	amino acid pathway
let-7f; 34a	FXYD2	FXYD domain containing ion transport regulator 2	0.66	−1.61	ion transport
34a	SHMT1	serine hydroxymethyl- transferase 1	0.75	−1.59	serine catabolism
181b	GPI	glucose phosphate isomerase	0.75	−1.52	glycolysis
34a	AMPD2	adenosine monophosphate deaminase 2	0.50	−1.47	energy metabolism (brain)
181b; 517*	HMBS	hydroxymethylbilane synthase	0.35	−1.24	heme biosynthesis
34a	LTF	lactotransferrin	0.15	−0.93	iron homeostasis
34a	CD151	CD151 antigen	0.59	−2.66	cell adhesion (sensory neuron)
181b	NDUFS3	NADH dehydrogenase Fe-S protein 3	0.08	−1.19	mitochondrial complex I

Cell survival program					
579	PSAP	prosaposin	0.79	−1.61	anti-apoptotic
34a	BIK	BCL2-interacting killer	0.54	−1.01	apoptosis

CNS and synapse					
let-7f; 34a	CRB3	crumbs homolog 3	0.39	−2.19	tight junction
34a	P2RY2	purinergic receptor P2Y, G-protein coupled, 2	0.51	−1.84	APP processing
579	RXRG	retinoid X receptor, gamma	0.49	−1.74	synaptic plasticity
34a	GFAP	glial fibrillary acidic protein	0.47	−1.65	myelination
181b; 517*	ACTG1	actin, gamma 1	0.77	−1.63	axon growth
34a	HNRPR	heterogeneous nuclear ribonucleoprotein R	0.45	−1.49	axon motor neuron

DNA repair					
181b; 200a	MCM3	minichromosome maintenance deficient 3	0.70	−1.68	DNA damage response
181b	MMS19L	MMS19-like (MET18 homolog)	0.59	−1.39	DNA repair

Inflammatory-immune response					
579	DDT	D-dopachrome tautomerase	0.76	−2.68	inflammation
let-7f; 34a	CHST12	carbohydrate sulfotransferase 12	0.79	−1.85	immune response
517*	LAMP2	lysosomal-associated membrane protein 2	0.50	−1.73	immune response
181b	SLA	Src-like-adaptor	0.53	−1.41	immune response

Injury response-redox homeostasis					
let-7f; 200a	HERPUD1	homocysteine-inducible, ubiquitin-likedomainmember1	0.41	−2.26	stress response
517*	HMGN2	high-mobility group nucleosomal binding domain 2	0.71	−1.71	oxidative stress
let-7f; 181b	TBPL1	TBP-like 1	0.41	−1.69	stress response
181b	HSF2	heat shock transcription factor 2	0.34	−1.64	stress response

Lipid metabolism					
let-7f	ELA3B	elastase 3B, pancreatic	0.53	−2.87	cholesterol biosynthesis
181b	FDXR	ferredoxin reductase	0.51	−1.85	cholesterol biosynthesis
let-7f; 34a	MECR	mitochondrial trans-2- enoyl-CoA reductase	0.50	−1.17	fatty acid metabolism

Proteasome-lysosome-transport					
579	UBE2M	ubiquitin-conjugating enzyme E2M	0.50	−2.13	ubiquitin cycle
181b	DNAJC7	DnaJ (Hsp40) homolog, subfamily C, member 7	0.76	−1.82	protein folding
181b	NDRG2	NDRG family member 2	0.43	−1.76	protein chaperone (misfolded)
579	SNX2	sorting nexin 2	0.34	−1.68	protein sorting

Signal transduction					
181b	AGGF1	Angiogenic factor with G patch and FHA domains 1	0.54	−1.44	signal transduction
181b	MAP3K6	mitogen-activated protein kinase kinase kinase 6	0.58	−0.93	signal transduction

Transcripion-translation					
34a	BTF3	basic transcription factor 3	0.45	−3.31	transcription
200a	DDX5	DEAD/H (Asp-Glu-Ala-Asp/His) box polypeptide 5	0.73	−2.34	spliceosome
34a	IRF1	interferon regulatory factor 1	0.59	−1.46	transcription
let-7f; 181b	TAF1A	TATA box binding protein- assoc. factor, RNApol I, A	0.58	−1.35	transcription (rRNA)

Unknown					
517*	MLF2	myeloid leukemia factor 2	0.71	−2.04	unknown
181b; 579	BEX2	Brain expressed X-linked 2	0.33	−1.89	unknown (brain)
let7f; 181b; 579	SSX2	synovial sarcoma, X breakpoint 2	0.46	−1.75	unknown
517*	ZNF691	zinc finger protein 691	0.14	−1.60	unknown
181b	RBMXL1	RNA binding motif protein, X-linked-like 1	0.42	−1.27	unknown
let-7f, 517*	OBFC1	oligonucleotide/oligosaccharide- bindingfoldcontaining1	0.55	−1.11	unknown

a Underlined gene symbols indicate similar down-regulation in AD affected brains (Maes et al. 2006).

## References

[b1-grsb-2007-263] Ambros V 2004 The functions of animal microRNAs Nature 431 350 5 15372042 10.1038/nature02871

[b2-grsb-2007-263] Babak T Zhang W Morris Q Blencowe BJ Hughes TR 2004 Probing microRNAs with microarrays: tissue specificity and functional inference Rna 10 1813 9 15496526 10.1261/rna.7119904 PMC1370668

[b3-grsb-2007-263] Bilen J Liu N Bonini NM 2006a A new role for microRNA pathways: modulation of degeneration induced by pathogenic human disease proteins Cell Cycle 5 2835 8 17172864 10.4161/cc.5.24.3579

[b4-grsb-2007-263] Bilen J Liu N Burnett BG Pittman RN Bonini NM 2006b MicroRNA pathways modulate polyglutamine-induced neurodegeneration Mol Cell 24 157 63 17018300 10.1016/j.molcel.2006.07.030

[b5-grsb-2007-263] Boehm M Slack F 2005 A developmental timing microRNA and its target regulate life span in C. elegans Science 310 1954 7 16373574 10.1126/science.1115596

[b6-grsb-2007-263] Chertkow H Bergman H Schipper HM Gauthier S Bouchard R Fontaine S Clarfield AM 2001 Assessment of suspected dementia Canadian Journal of Neurological Sciences 28 Suppl 1 S28 41 11237308 10.1017/s0317167100001189

[b7-grsb-2007-263] Didiano D Hobert O 2006 Perfect seed pairing is not a generally reliable predictor for miRNA-target interactions Nat Struct Mol Biol 13 849 51 16921378 10.1038/nsmb1138

[b8-grsb-2007-263] Folstein MF Folstein SE Mchugh PR 1975 “Mini-mental state”. A practical method for grading the cognitive state of patients for the clinician J Psychiatr Res 12 189 98 1202204 10.1016/0022-3956(75)90026-6

[b9-grsb-2007-263] Hammond SM 2006 RNAi, microRNAs, and human disease Cancer Chemother Pharmacol 58 Suppl 7 63 8 10.1007/s00280-006-0318-2 17093929

[b10-grsb-2007-263] Hsiung GY Sadovnick AD Feldman H 2004 Apolipoprotein E epsilon4 genotype as a risk factor for cognitive decline and dementia: data from the Canadian Study of Health and Aging Cmaj 171 863 7 15477624 10.1503/cmaj.1031789 PMC522651

[b11-grsb-2007-263] Jison ML Munson PJ Barb JJ Suffredini AF Talwar S Logun C Raghavachari N Beigel JH Shelhamer JH Danner RL Gladwin MT 2004 Blood mononuclear cell gene expression profiles characterize the oxidant, hemolytic, and inflammatory stress of sickle cell disease Blood 104 270 80 15031206 10.1182/blood-2003-08-2760 PMC5560446

[b12-grsb-2007-263] Kamboh MI 2004 Molecular genetics of late-onset Alzheimer’s disease Ann Hum Genet 68 381 404 15225164 10.1046/j.1529-8817.2004.00110.x

[b13-grsb-2007-263] Keller JN 2006 Interplay Between Oxidative Damage, Protein Synthesis, and Protein Degradation in Alzheimer’s Disease Journal of Biomedicine and Biotechnology, Volume 2006 1 3 10.1155/JBB/2006/12129 PMC1510934 17047298

[b14-grsb-2007-263] Krek A Grun D Poy MN Wolf R Rosenberg L Epstein EJ Macmenamin P Da Piedade I Gunsalus KC Stoffel M Rajewsky N 2005 Combinatorial microRNA target predictions Nat Genet 37 495 500 15806104 10.1038/ng1536

[b15-grsb-2007-263] Lacelle C Riol H Xu S Tang YJ Wang YS Chuang YL Lin HS Chang MC Liang J Wang E 2002 Blood-sample processing for the study of age-dependent gene expression in peripheral blood mononuclear cells J Gerontol A Biol Sci Med Sci 57 B285 7 12084799 10.1093/gerona/57.7.b285

[b16-grsb-2007-263] Lee RC Feinbaum RL Ambros V 1993 The C. elegans heterochronic gene lin-4 encodes small RNAs with antisense complementarity to lin-14 Cell 75 843 54 8252621 10.1016/0092-8674(93)90529-y

[b17-grsb-2007-263] Lim LP Lau NC Garrett-Engele P Grimson A Schelter JM Castle J Bartel DP Linsley PS Johnson JM 2005 Microarray analysis shows that some microRNAs downregulate large numbers of target mRNAs Nature 433 769 73 15685193 10.1038/nature03315

[b18-grsb-2007-263] Lu J Getz G Miska EA Alvarez-Saavedra E Lamb J Peck D Sweet-Cordero A Ebert BL Mak RH Ferrando AA Downing JR Jacks T Horvitz HR Golub TR 2005 MicroRNA expression profiles classify human cancers Nature 435 834 8 15944708 10.1038/nature03702

[b19-grsb-2007-263] Lukiw WJ 2007 Micro-RNA speciation in fetal, adult and Alzheimer’s disease hippocampus Neuroreport 18 297 300 17314675 10.1097/WNR.0b013e3280148e8b

[b20-grsb-2007-263] Maes OC Xu S Yu B Chertkow HM Wang E Schipper HM 2006 Transcriptional profiling of Alzheimer blood mononuclear cells by microarray Neurobiol Aging10.1016/j.neurobiolaging.2006.08.004 16979800

[b21-grsb-2007-263] Markesbery WR Lovell MA 2006 DNA oxidation in Alzheimer’s disease Antioxid Redox Signal 8 2039 45 17034348 10.1089/ars.2006.8.2039

[b22-grsb-2007-263] Mckhann G Drachman D Folstein M Katzman R Price D Stadlan EM 1984 Clinical diagnosis of Alzheimer’s disease: report of the NINCDS-ADRDA Work Group under the auspices of Department of Health and Human Services Task Force on Alzheimer’s Disease Neurology 34 939 44 6610841 10.1212/wnl.34.7.939

[b23-grsb-2007-263] Ouellet DL Perron MP Gobeil LA Plante P Provost P 2006 MicroRNAs in Gene Regulation: When the Smallest Governs It All J Biomed Biotechnol 2006 69616 17057368 10.1155/JBB/2006/69616 PMC1559927

[b24-grsb-2007-263] Park W Li J Song R Messing J Chen X 2002 CARPEL FACTORY, a Dicer homolog, and HEN1, a novel protein, act in microRNA metabolism in Arabidopsis thaliana Curr Biol 12 1484 95 12225663 10.1016/s0960-9822(02)01017-5 PMC5137372

[b25-grsb-2007-263] Pasinetti GM 2001 Use of cDNA Microarray in the Search for Molecular Markers Involved in the Onset of Alzheimer’s Disease Dementia Journal of Neuroscience Research 65 471 476 11550214 10.1002/jnr.1176

[b26-grsb-2007-263] Puchades M Hansson SF Nilsson CL Andreasen N Blennow K Davidsson P 2003 Proteomic studies of potential cerebrospinal fluid protein markers for Alzheimer’s disease Brain Res Mol Brain Res 118 140 6 14559363 10.1016/j.molbrainres.2003.08.005

[b27-grsb-2007-263] Ruvkun G Wightman B Ha I 2004 The 20 years it took to recognize the importance of tiny RNAs Cell 116 S93 6 2 p following S96 15055593 10.1016/s0092-8674(04)00034-0

[b28-grsb-2007-263] Scherzer CR Offe K Gearing M Rees HD Fang G Heilman CJ Schaller C Bujo H Levey AI Lah JJ 2004 Loss of apolipoprotein E receptor LR11 in Alzheimer disease Arch Neurol 61 1200 5 15313836 10.1001/archneur.61.8.1200

[b29-grsb-2007-263] Selkoe DJ 1991 The molecular pathology of Alzheimer’s disease Neuron 6 487 98 1673054 10.1016/0896-6273(91)90052-2

[b30-grsb-2007-263] Shan X Lin CL 2006 Quantification of oxidized RNAs in Alzheimer’s disease Neurobiol Aging 27 657 62 15979765 10.1016/j.neurobiolaging.2005.03.022

[b31-grsb-2007-263] Sood P Krek A Zavolan M Macino G Rajewsky N 2006 Cell-type-specific signatures of microRNAs on target mRNA expression Proc Natl Acad Sci USA 103 2746 51 16477010 10.1073/pnas.0511045103 PMC1413820

[b32-grsb-2007-263] Strauss WM Chen C Lee CT Ridzon D 2006 Nonrestrictive developmental regulation of microRNA gene expression Mamm Genome 17 833 40 16897339 10.1007/s00335-006-0025-7

[b33-grsb-2007-263] Subramanian A Tamayo P Mootha VK Mukherjee S Ebert BL Gillette MA Paulovich A Pomeroy SL Golub TR Lander ES Mesirov JP 2005 Gene set enrichment analysis: A knowledge-based approach for interpreting genome-wide expression profiles Proc Natl Acad Sci USA 102 15545 15550 16199517 10.1073/pnas.0506580102 PMC1239896

[b34-grsb-2007-263] Wang E Lacelle C Xu S Zhao X Hou M 2002 Designer microarrays: from soup to nuts J Gerontol A Biol Sci Med Sci 57 B400 5 12403795 10.1093/gerona/57.11.b400

